# Anti-BCMA Immunotoxins: Design, Production, and Preclinical Evaluation

**DOI:** 10.3390/biom10101387

**Published:** 2020-09-29

**Authors:** Tapan K. Bera

**Affiliations:** Laboratory of Molecular Biology, Center for Cancer Research, NCI, NIH, Bethesda, MD 20892, USA; BeraT@mail.nih.gov

**Keywords:** mAb BM306, LMB-70, ABD fusion protein, H929 cells

## Abstract

Multiple myeloma (MM) is a B-cell malignancy that is incurable for a majority of patients. B-cell maturation antigen (BCMA) is a lineage-restricted differentiation protein highly expressed in multiple myeloma cells but not in other normal tissues except normal plasma B cells. Due to the restricted expression and being a cell surface membrane protein, BCMA is an ideal target for immunotherapy approaches in MM. Recombinant immunotoxins (RITs) are a novel class of protein therapeutics that are composed of the Fv or Fab portion of an antibody fused to a cytotoxic agent. RITs were produced by expressing plasmids encoding the components of the anti-BCMA RITs in *E. coli* followed by inclusion body preparation, solubilization, renaturation, and purification by column chromatography. The cytotoxic activity of RITs was tested in vitro by WST-8 assays using BCMA expressing cell lines and on cells isolated from MM patients. The in vivo efficacy of RITs was tested in a xenograft mouse model using BCMA expressing multiple myeloma cell lines. Anti-BCMA recombinant immunotoxins are very effective in killing myeloma cell lines and cells isolated from myeloma patients expressing BCMA. Two mouse models of myeloma showed that the anti-BCMA immunotoxins can produce a long-term complete response and warrant further preclinical development.

## 1. Introduction

Multiple myeloma is a cancer of plasma cells that originates in the bone marrow, where normal plasma cells are produced. The precise etiology that transform normal antibody producing plasma cells into malignant multiple myeloma (MM) is not clear. Globally there were over 138,000 new cases and 98,000 deaths from multiple myeloma in 2016 [1]. A recent report from the American Cancer Society suggests that in the USA alone there will be 32,270 new cases of MM in 2020 and the estimated number of deaths from this disease will be 12,830 [2]. Recent advances in antibody based therapeutic research have led to the advent of promising treatment options for multiple myeloma. The development of an anti-CD38 antibody as a therapeutic regimen alters the treatment landscape for multiple myeloma. CD38 was identified as a multifunctional transmembrane type II glycoprotein almost 40 years ago and is predominantly expressed on terminally differentiated plasma cells [3]. CD38 is highly expressed in malignant plasma cells, making it an attractive therapeutic target antigen. The anti-CD38 monoclonal antibody (mAb), daratumumab, is currently approved by the FDA for the treatment of relapsed/refractory multiple myeloma both as monotherapy and in combination with other chemotherapeutic drugs. Another anti-CD38 mAb, isatuximab, is currently in late stage clinical development for the treatment multiple myeloma [4]. Although regulatory approval of two monoclonal antibodies, one targeting CD38 (daratumumab) and the other (elotuzumab) targeting a member of the Signaling Lymphocyte Activation Molecule Family protein in 2015 was an important advancement for the immuno-based therapy of MM, the expression of both target antigens in other essential tissues restrict their clinical application. Identification of B cell maturation antigen (BCMA), that is selectively expressed in mature B-lymphocytes and in almost all MM cells from patients showed some promise for an effective immune-based therapy to treat this disease.

This review discusses the characteristics of BCMA as an attractive immune-based therapeutic target and various treatment strategies targeting BCMA that are under clinical development. Among various anti-BCMA therapeutic strategies, this review will specifically address the current developmental status of anti-BCMA immunotoxin as a potential therapeutic option for the treatment of relapsed and refractory multiple myeloma.

## 2. BCMA as Therapeutic Target

**Figure 1:**
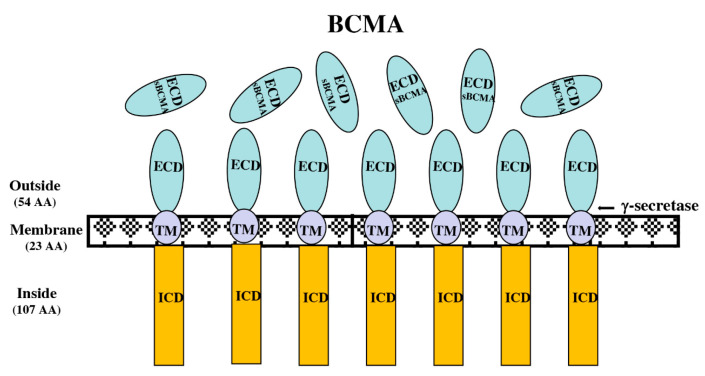
Schematics showing B-cell maturation antigen (BCMA) protein. BCMA is a type III transmembrane protein with no signal sequence at the amino-terminus. Extracellular domain (ECD) of BCMA comprises of 54 amino acids and shed as soluble BCMA (sBCMA) after cleaved by γ-secretase. The transmembrane domain (TM) and the intracellular domain (ICD) of BCMA contain 23 and 107 amino acid residues, respectively.

BCMA, also known as tumor necrosis factor receptor superfamily member 17 (TNFRSF17), was cloned and characterized as a mature B-cell marker over 25 years ago [5]. BCMA is a single pass type III outer membrane glycoprotein with a short (54 amino acid) extracellular domain without a signal-sequence (Figure 1). 

BCMA and two other related superfamily members, BAFF-R and TACI, tightly regulate B-cell homeostasis as well as differentiation into plasma cells. Two cognate ligands, BAFF and APRIL, bind to these three functionally related receptors to support long term survival of B cells at different stages of development. However, the reported binding affinities of each ligand for each receptor varies significantly, suggesting a delicate regulation of B-cell homeostasis. B-cells are generated in the bone marrow and go through sequential developmental stages to develop from hematopoietic stem cells to mature B-cells. After leaving bone marrow, B-cells migrate to secondary lymphoid organs and undergo further differentiation. During this process of maturation, which takes 1–2 weeks, expression of several cell surface receptors and adhesion molecules play a critical role in B-cell proliferation and differentiation. Mice lacking the BCMA gene have no obvious impact on B cell life span or function [6]. Flow cytometry analysis of bone marrow cells from BCMA knock out mice showed no impairment of B220^−^ IgM^+^ pre-B as well as B220^+^ IgM^+^ immature B cell populations compared to bone marrow cells from wild-type mice, suggesting BCMA does not play any role in early B cell development. The composition of B-cell subtypes such as marginal zone B-cells in the spleens of BCMA deficient mice has also remained unchanged compared to its wild-type counterpart, indicating BCMA plays no role in B-cell expansion in the spleen. Total number of B-cells in bone marrow and other secondary lymphoid organs such as spleen, lymph nodes, and the peritoneal cavity of BCMA knock out mice is very comparable to those of wild type control mice. Studies on BCMA deficient mice also showed normal humoral immune responses when challenged with exogenous immunogens. The serum concentrations of immunoglobulins (IgA, IgE, IgM, IgG1, IgG2a, IgG2b, and IgG3) in BCMA mutant mice are all within normal range suggesting a functional immune system [6]. However, later studies showed that BCMA is required for the survival of long-lived bone marrow plasma cells [7]. BCMA is selectively expressed on the surface of differentiated plasma cells (PC) but not on hematopoietic stem cells, memory, or naïve B cells. An elegant gene expression profiling study by Seckinger et al. using hundreds of samples from newly diagnosed and relapse refractory MM (RRMM) patients showed that BCMA is universally and specifically expressed in all tested malignant plasma cells [8]. In this study BCMA expression was investigated by gene expression profiling using RNA isolated from malignant plasma cells (PC) from 712 samples of previously untreated or relapsed myeloma patients. In addition, RNA deep sequencing was performed and analyzed on 263 RNA samples isolated from patients with highly proliferative disease or with high-risk features based on chromosomal aberrations. The surface expression of BCMA antigen was validated on malignant myeloma cells by multidimensional flow cytometry. Although BCMA is expressed in normal plasma B cells, the BCMA antigen density on malignant plasma cells is significantly higher than in other cell types in normal bone marrow cell components as evident by flow cytometry analysis. Although BCMA expression is found on virtually all primary and relapsed refractory multiple myeloma specimens, it is also expressed in other hematologic cancers. BCMA expression is frequently found in patients with chronic lymphocytic lymphoma (CLL), non-Hodgkins lymphoma (NHL), and patients with B-ALL and T-ALL. Studies have shown that membrane bound BCMA is cleaved by gamma secretase [9]. The elevated levels of serum BCMA (sBCMA) have been reported in patients with multiple myeloma and chronic lymphocytic leukemia. It is not clear if the serum BCMA has any role in B-cell homeostasis or PC differentiation. However, additional studies show that symptomatic myeloma patients with high tumor burden had the highest sBCMA level in their blood, followed by asymptomatic myeloma patients. Serum BCMA levels can be predictive of treatment effectiveness and overall survival of multiple myeloma patients [10]. Because of its selective robust expression in almost all malignant plasma cells from patients with primary or relapsed refractory multiple myeloma, but not in other essential vital organs, BCMA has become an attractive immune-based drug target for the treatment of multiple myeloma [11]. BCMA has been investigated as the target in various immunotherapeutic strategies including BCMA-directed mAb [12], antibody drug conjugate [13], chimeric antigen receptor therapy [14], and therapy with bispecific T-cell engagers (BiTEs) [15]. There are several promising clinical trials in progress with various payloads targeting BCMA [16].

## 3. BCMA-Directed mAb

The first anti-BCMA mAb developed as a therapeutic (SG1) was generated by Seattle Genetics in 2007 to treat multiple myeloma patients. SG1 was developed as a naked antibody to induce ADCC dependent cell killing. SG1 was also investigated as an antibody drug conjugate with a potent cytotoxic drug monomethyl auristatin F [12]. The preclinical data for both SG1 as naked antibody or as an antibody drug conjugates (ADC) was very encouraging, but no further studies for clinical development have been reported.

## 4. Anti-BCMA Drug Conjugate

The leading anti-BCMA drug conjugate GSK2857916 is a humanized IgG1 mAb, with a sub nanomolar affinity to BCMA, that was conjugated with monomethyl auristatin F (MMAF), via a protease resistant, stable, non-cleavable linker. The anti-mitotic drug MMAF is a microtubule disrupting agent that inhibits cell division by blocking tubulin polymerization. After binding to the cell surface via anti-BCMA antibody interaction, GSK2857916 gets into the cell rapidly and releases the anti-mitotic drug MMAF inside the cell and induces cell death. GSK2857916 was investigated intensively and showed robust in vitro activity on all tested CD138+ and BCMA+ myeloma cell lines as well as cells isolated from multiple myeloma patients [17]. The antibody also triggers an anti-tumor immune response by natural killer (NK) cells, and macrophage mediated phagocytosis [18]. In clinical trials GSK2857916 is well-tolerated and effective in heavily pre-treated MM patients with grade 3 or 4 toxicity, mostly of hematological nature. Another frequent adverse effect of GSK2857916 was corneal toxicity including eye dryness, pain, and inflamed cornea. Based on the promising first-in-human clinical trial results [19], the FDA granted GSK2857916 a breakthrough therapy designation to treat relapsed refractory myeloma patients who failed at least three prior lines of therapy. A phase1/2 clinical trial was conducted to evaluate the role of GSK2857916 in combination with other standard of care regimens for the treatment of relapsed refractory myeloma [20]. In August of 2020, FDA approved GSK2857916 (belantamab mafodotin) to treat adult patients with RRMM who have received at least four prior therapies including a proteosome inhibitor, anti-CD38 mAb and an immunomodulatory agent.

Anti-BCMA drug conjugate MEDI2228, is also in advanced phase of clinical evaluation. It is a novel ADC comprised of a humanized anti-BCMA antibody conjugated to DNA-damaging drug pyrrolobenzodiazepine by a cleavable linker [21,22]. MEDI2228 was evaluated in preclinical models and shown to be very effective in vitro on BCMA-expressing cell lines as well as on primary relapsed/refractory myeloma cells from patients. In a mouse model, complete elimination of tumors was seen when MEDI2228 was given as a single agent. In addition, MEDI2228 in combination with bortezomib acted synergistically to decrease cell viability of drug-resistant myeloma cells in vitro. Cotreatment of both agents also improved survival of mice carrying myeloma tumors [23]. Based on the encouraging preclinical data, a phase 1 clinical trial of MEDI2228 as single agent has begun in relapsed/refractory myeloma patients.

## 5. BCMA Directed CAR-T Cell

T cells expressing a chimeric antigen receptor (CAR-T) is currently one of the leading therapeutic interventions for the treatment of cancer. CAR-T cells equipped with a genetically altered receptor that targets a specific cancer antigen have been employed as adoptive immunotherapy and has become one of the most promising treatment options for many cancer types. CAR-T cell therapy received a huge boost in the pharmaceutical industry as a successful therapeutic option to treat hematologic malignancies due to the recent FDA approval of Kymriah and Yescarta; the first two commercial CAR-T products for the treatment of acute lymphocytic lymphoma and large B cell lymphoma. Like other targeted therapy regimens, specificity of target antigen expression is the most important criteria for a successful CAR-T cell against a cancer type. Due to the specific robust expression in MM cells and virtually no expression in any vital organs, BCMA is an ideal target antigen for CAR-T therapy for multiple myeloma. Several promising BCMA-directed CAR-T products are in different phases of clinical trials for the treatment of MM patients. Among the leading candidates, BB2121, developed by Bluebird Bio/Celgene, is at the forefront with the most advanced clinical development data. BB2121 is comprised of the Fv fragment of a murine anti-BCMA antibody 11D5-3 in a lentivirus vector backbone and contains a 4-1BB costimulatory domain [24]. The efficacy of BB2121 was evaluated with relapsed refractory myeloma patients in the multicenter phase 1 trial. Based on the promising evaluation of the dose escalation study, BB2121 was advanced to a breakthrough therapy designation by the FDA and a global multicenter phase 2 clinical trial is currently in progress. The second most advanced CAR-T cell construct (LCAR-B38M) in development consists of a dual epitope specific Fv targeting BCMA antigen and is being developed by Legend Biotech/Johnson & Johnson to treat relapsed refractory multiple myeloma [25]. Several other promising anti-BCMA CAR-T constructs are currently in preclinical and clinical evaluation.

## 6. Anti-BCMA BiTEs

Bispecific T-cell engagers (BiTEs) are a new group of immunotherapeutic reagents that contain two different domains, one targeting the cancer specific antigen and other to CD3e on T cells. This bispecific molecule brings the cancer cells closer to the T cell to facilitate the cytolytic effect of the T cell selective to the cancer cell. Several anti-BCMA BiTEs are being investigated or under development as a potential therapeutic application for the treatment of patients with relapsed refractory multiple myeloma. BI 836909 is an anti-BCMA BiTEs developed by Amgen to target BCMA antigen on the surface of myeloma cells. Hipp et al. reported that anti-BCMA BiTE BI836909 induced selective cell lysis of MM cell lines in vitro and can deplete H929 myeloma cells in a subcutaneous xenograft mouse model [26]. In a co-culture in vitro study with unstimulated peripheral blood mononuclear cell (PBMCs) and BCMA positive myeloma cell lines, BI 836909 induced dose-dependent robust tumor cell lysis of the myeloma cell lines with EC90 values ranging from 16–810 pg/mL. Anti-BCMA BiTEs directed killing is specific for BCMA expressing cells as the viability of BCMA-negative cells were not affected. BI 836909 has been tested in both subcutaneous and orthotopic xenograft models using human T cells and two BCMA-expressing myeloma cell lines. In both models BI 836909 was proven to be highly effective, and significantly prolonged survival when treated at doses that are non-toxic to the mice. In toxicity studies, BI 836909 was reported to be well tolerated by cynomolgus macaques with no obvious side effects. Based on the preclinical data, BI 836909 is currently being evaluated in a phase I dose escalation trial with relapsed/refractory MM patients. 

Similar to BiTEs targeting BCMA, a tri-specific T cell activating (TriTAC) molecule has been evaluated. HPN217 is a TriTAC consisting of a single domain anti-BCMA antibody linked to a single domain antibody that binds human serum albumin followed by an anti-CD3e single chain Fv. The activity of HPN217 was evaluated in vitro and in mice by T cell-dependent cell lysis assay and has been advanced to a first-in-human clinical trial with relapsed/refractory multiple myeloma patients [27]. 

Several other approaches have been investigated in early phase clinical trials as a potential therapeutic modality targeting BCMA for the treatment of MM [28,29,30]. Among them Descartes-08, RNA-generated anti-BCMA CD8 CAR T cells, developed by Cartesian Therapeutics showed promising results in a mouse model of disseminated MM and advanced to clinical trial in patients with RRMM [28]. A recent review by Cho et al. highlighted important preclinical and ongoing clinical studies of various BCMA-targeted therapeutic modalities for the treatment of MM [31].

## 7. Recombinant Immunotoxin

Immunotoxins are a class of bifunctional chimeric protein molecules composed of a domain, usually an antibody, that can bind to a cell in a target specific manner, and the other a cytotoxic domain [32]. Immunotoxins get their cell killing properties from the toxin and their specificity from the antibody domain. Two well characterized toxins used to generate immunotoxin for therapeutic applications are *Pseudomonas* exotoxin A (PE) and diphtheria toxin (DT). First generation immunotoxins were made of a whole antibody chemically coupled with a toxin moiety, after deleting the native cell binding domain of the toxin. The process of generating enough immunotoxin for preclinical or clinical studies using chemical coupling methods is complicated and expensive. Recombinant immunotoxins (RITs) are composed of an antibody variable fragment fused to a portion of the bacterial toxin *Pseudomonas* exotoxin A (PE). RITs are generated by recombinant DNA technology. RITs can be modified easily by genetic engineering to enhance their properties and can be produced in bacteria in large quantities in a relatively less expensive manner than the chemical conjugates. Since the toxin domain of the immunotoxin is of bacterial origin, it is very immunogenic and is an obstacle to use as a therapeutic in patients. However, the immunotoxin has several properties that are advantageous over the other commonly used anti-cancer therapeutics especially antibody drug conjugates (ADC). Immunotoxins exerts their cell killing properties by attacking the cellular protein synthesis machinery. Upon binding to the cell surface target, an antigen-immunotoxin complex enters into the endosomal compartment followed by transfer into the cytosol by retrograde transport. The toxin moiety of the immunotoxin then inactivates elongation factor 2 (EF2) in the cytosol by ADP-ribosylation and inhibits protein synthesis. Inactivation of EF2 triggers a cascade of events leading to disruption of delicate cellular machinery and induces apoptosis and cell death. Due to the unique killing mechanism and unlike commonly used chemotherapeutics, immunotoxins are effective on quiescent cells. In addition, the immunotoxin can be used in combination with other approved therapeutics due to their nonoverlapping toxicity profile. Several RITs are in preclinical development or in clinical trials [33]. Moxetumomab pasudotox (Lumoxiti, Innate Pharma Inc., Rockville, MD, USA), which targets CD22 on B cell malignancies, has a very high response rate in hairy cell leukemia, has produced complete and durable responses in many patients and was approved by the FDA for treatment of relapsed or refractory hairy cell leukemia patients [34]. An immunotoxin targeting mesothelin (SS1P, followed by LMB-100) has also caused major tumor regressions in patients with chemotherapy-resistant malignant mesothelioma [33].

## 8. RIT Targeting BCMA

To develop immunotoxins targeting BCMA, several monoclonal antibodies (mAb) were generated by immunizing mice with a recombinant BCMA protein using hybridoma techniques. Mice were initially immunized with BCMA-rFc (rabbit fragment crystallizable region) fusion protein in adjuvant intraperitoneally for the first immunization, and subsequently immunized 5 times with BCMA-rFc without adjuvant. High antibody titers (1:10,000) against the BCMA-expressing 293T cells were detected in the sera from mice immunized with BCMA-rFc. The mice with high-titer were given a final boost by injecting BCMA-rFc with adjuvant intraperitoneally, and 3 days later, the spleens were fused with P3U1 myeloma cells. To examine the cross-reactivity of the anti-BCMA mAbs with other TNFR superfamily members, the reactivity of each mAb at a saturated concentration (4 μg/mL) was tested on transfected 293T cells expressing native TNFRs (BCMA, TACI or BAFFR) by FACS. Out of several high affinity mAbs, BM306 was selected for further development based on high-affinity binding (<1 × 10^–10^ M) to BCMA antigen on the cell surface and no cross reactivity to closely-related TNFRs (TACI or BAFFR) [35]. 

**Figure 2:**
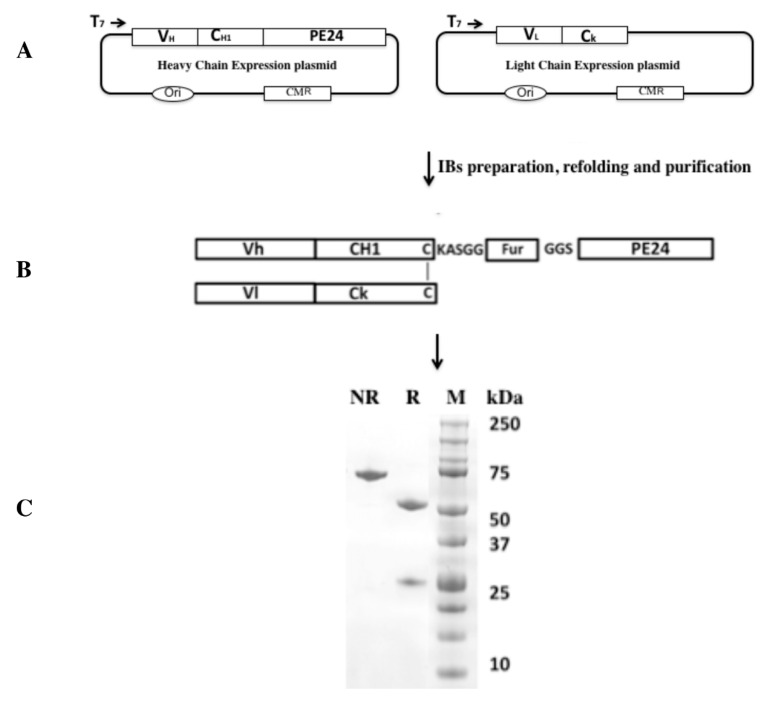
Production scheme of an anti-BCMA Fab immunotoxin. (**A**) Schematics showing expression plasmids encoding the heavy chain linked to toxin and the second encoding the light chain. Both plasmids contain an inducible T7 transcription promoter and a chloramphenicol antibiotic resistance gene (CM^R^) as selectable marker. (**B**) Schematics of correctly folded Fab immunotoxin protein. (**C**) Analytical PAGE gel showing the quality of purified Fab immunotoxin. Lanes: NR, protein analyzed under non-reducing condition; R, protein analyzed under reducing condition; M, molecular weight standards.

BM306 mAb is IgG1 isotype; the antibody heavy and the light chains (Fvs) were cloned from the BM306 expressing hybridomas using IgG1 isotype-specific oligo primers. To generate a Fab-immunotoxin, two expression plasmids for BM306 mAb were developed (Figure 2). 

**Figure 3:**
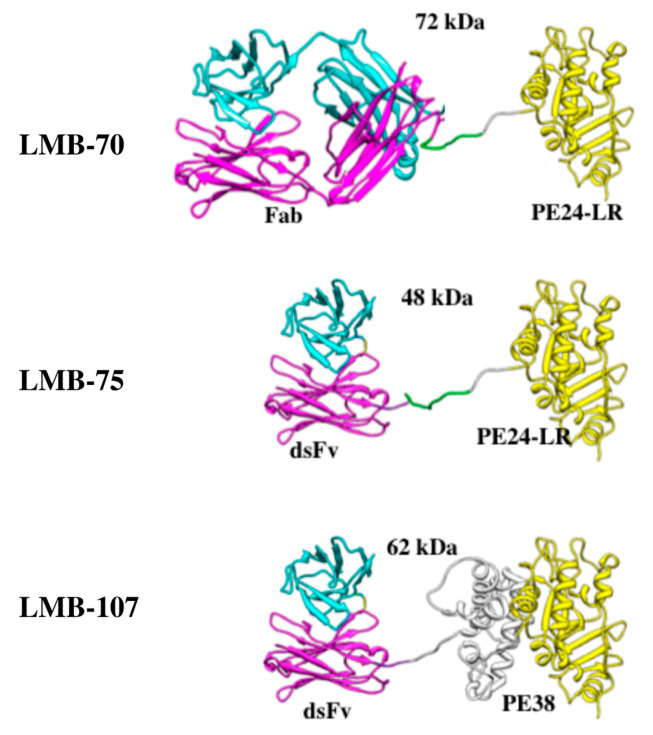
Ribbon drawing of ant-BCMA immunotoxin variants. The light chain (cyan) and the heavy chain (magenta) were modeled using X-ray structure data. Domains II and III of PE (Pseudomonas exotoxin) data were taken from 1hkl.pdb and are represented in grey and yellow, respectively. The linker between the Fv and the toxin containing the furin cleavage site is shown in green. Length and conformation of the linker were chosen arbitrarily. The predicted molecular weights of LMB-70, LMB-75, and LMB-107 are 72 kDa, 48 kDa, and 62 kDa, respectively.

**Figure 4:**
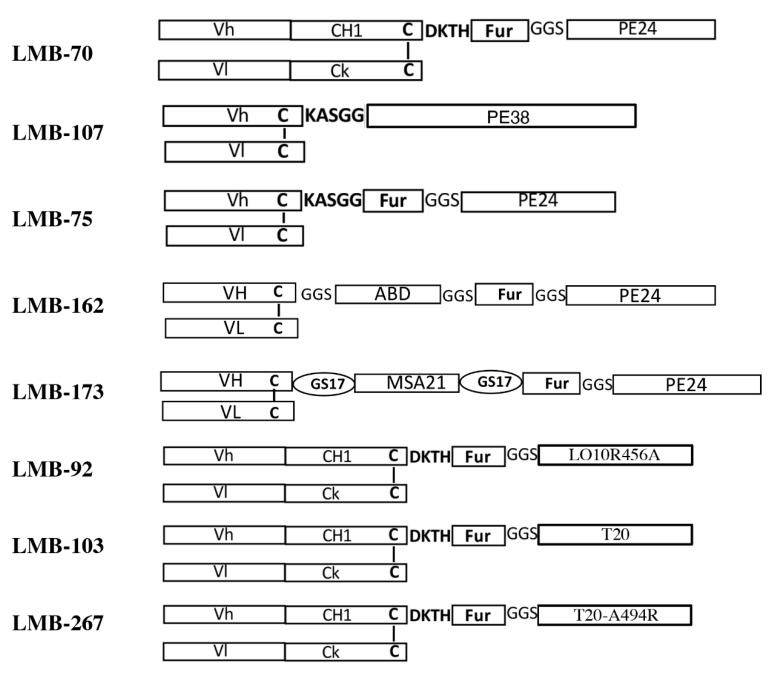
Schematics showing anti-BCMA immunotoxin variants described in this review. LMB-70: BM306-Fab linked to 24 kDa fragment of PE; LMB-107: BM306-Fv linked to 38 kDa fragment of PE; LMB-75: BM306-Fv linked to 24 kDa fragment of PE; LMB-162: BM306-Fv linked to 54 amino acid fragment albumin binding domain from Streptococcus followed by 24 kDa fragment of PE; LMB-173: BM306-Fv linked to single domain anti-albumin Llama antibody followed be 24 kDa fragment of PE; LMB-92: BM306-Fab fragment linked to less-immunogenic B-cell epitope-removed version of PE; LMB-103: BM306-Fab fragment linked to less-immunogenic T-cell epitope-removed version of PE; LMB-267: BM306-Fab fragment linked to less-immunogenic T-cell epitope-removed version of PE with Ala to Arg mutation at residue 294. Fur: Furin cleavage sequence of PE.

One construct encodes Fd (heavy chain of antigen binding fragment) chain containing the Vh (variable loop of heavy chain) and the CH1 sequence of IgG1. The Fd chain fragment was fused with a modified version of PE in an immunotoxin expression vector. The second plasmid encodes the light chain containing the Vl (variable loop of light chain) sequence of the mAb and the Ck (constant domain of kappa light chain) constant region of light chain. Both constructs are expressed separately as inclusion bodies in *E. coli*, refolded in vitro by redox shuffling, then purified (Figure 2) using ion exchange and sizing columns [36]. Ribbon drawings of anti-BCMA immunotoxin variants are shown in Figure 3 and schematics of anti-BCMA immunotoxin variants under preclinical development are shown in Figure 4. 

LMB-70 activity was tested on several BCMA positive myeloma cell lines in vitro. The activity of LMB-70 corelates with the expression of BCMA and ranged from 1 ng/mL (most sensitive cell line, H929) to 20 ng/mL (on LP1 cell line) [35]. Cell killing efficacy of the immunotoxins are greatly dependent on the cellular uptake and internalization of the toxin bound cell surface antigen. When tested in vitro on the H929 cell line, LMB-70 appears to be internalized very rapidly as only 10 min of immunotoxin exposure was sufficient to induce complete cell killing [25]. Cytotoxicity was associated with induction of the apoptotic pathway as evident by decreased levels of anti-apoptotic protein MCL-1 and BCL-XL as well as cleavage of caspases 3, 8, and 9. The activity of LMB-70 was measured on primary cells obtained from patients with multiple myeloma. Bone marrow mononuclear cells (BMMNCs) from seven patients with active disease who had considerable numbers of myeloma plasma cells in the BM were treated with LMB 70. The BMMNCs of all seven patient samples tested were killed efficiently by LMB-70 with IC_50_ values ranging from 4 to 17 ng/mL ng/mL [35]. 

Based on the encouraging in vitro cytotoxicity data, the in vivo efficacy of LMB-70 immunotoxin was tested in a mouse subcutaneous xenograft model using the most sensitive H929 cell line. When SCID mice bearing H929 xenografts were treated with 1.5 mg/kg QODX5 doses of LMB-70, the growth of the subcutaneous tumors was delayed but did not produce any complete responses [35]. The LMB-70 treated tumors grew back once the treatment was stopped. However, the immunotoxin LMB-11 targeting CD22, which is very similar to LMB-70 with an IC_50_ of 1 ng/mL on cells expressing CD22 in vitro, produced complete responses when tested in mouse xenograft models with a dose of 1.5 mg/kg QODX5 [37]. The basis of the incomplete in vivo responses of LMB-70 on this subcutaneous xenograft model is not due to antigen loss, and cells isolated from treated tumors are fully responsive when returned to cell culture [38]. The molecular mechanism responsible for the inefficacy of LMB-70 on this subcutaneous xenograft tumor warrants further investigation.

Nab-paclitaxel (Abraxane®, Celgene corporation, Summit, NJ, USA), a modified version of paclitaxel, is an anti-microtubule agent that has been studied as a chemotherapeutic agent to treat various types of solid tumors including multiple myeloma [39]. Abraxane has synergistic antitumor activity in a pancreatic tumor model when combined with immunotoxin RG7787 targeting mesothelin [40]. LMB-70 in combination with Abraxane shows remarkable synergy and induced complete responses in a subcutaneous H929 xenograft model of multiple myeloma [35].

Multiple myeloma is a plasma cell cancer that originates and proliferates in the bone marrow of myeloma patients. The activity of anti-BCMA immunotoxin LMB-70 was investigated in a more relevant bone marrow mouse model. To generate the bone marrow mouse model, the myeloma cell lines were engineered by transfecting lentivirus expressing GFP-Luciferase genes [41]. When myeloma-Luc cells were injected intravenously into the tail vein of NSG mice, cells grew and populated the bone marrow as assessed by bioluminescence imaging and showed a growth pattern similar to those in myeloma patients. 

**Figure 5:**
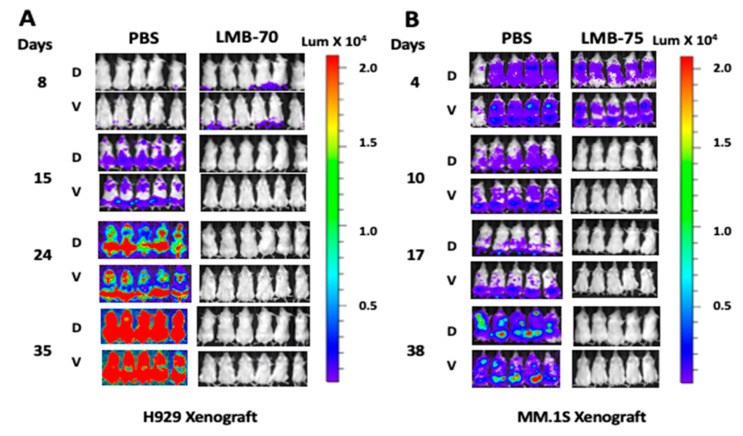
Efficacy of anti-BCMA immunotoxin in mouse bone marrow xenograft models. H929-Luc-GFP (**A**) and multiple myeloma (MM). 1S-Luc-GFP (**B**) cells were injected IV into NSG mice and were treated IV with PBS or 1.5 mg/kg immunotoxin QOD × 5. Bioluminescence imaging was used to assess tumor burden. Dorsal (D) and ventral (V) images are shown for each mouse.

Anti-BCMA immunotoxin LMB-70 was tested for anti-tumor efficacy in the mouse bone marrow xenograft model with two myeloma cell lines. In one model, H929-luc cells were used and the immunotoxin was given IV every other day starting on day 8 after cell injection [41]. In contrast to the subcutaneous xenograft model, LMB-70 produced a complete response in all treated mice when tested in mouse bone marrow xenograft harboring H929-luc myeloma cells with a dose of 1.5 mg/kg QODX5 (Figure 5).

In a second model with MM.1S-luc bone marrow xenograft, LMB-75, an Fv variant of anti-BCMA immunotoxin (Figure 4) showed complete responses in all treated mice with similar dose of 1.5 mg/kg QODX5 (Figure 5). The result from these two xenograft bone marrow models suggested that anti-BCMA immunotoxins are very effective in eliminating BCMA expressing myeloma cells from the bone marrow of mice and represent a potential treatment option to effectively eliminate the myeloma cell in humans.

## 9. Anti-BCMA IT with Improved Properties

Efforts have been made to improve the properties of anti-BCMA immunotoxins. The pharmacokinetic properties of the immunotoxin molecule plays an important role for the in vivo efficacy of RIT. Often, a short half-life of immunotoxin molecules restrict their efficacy because the immunotoxin gets cleared from the circulation very quickly and does not have enough time to target the cancer cell, particularly for solid tumors, which have penetration limitations. The half-life of LMB-70 (Fab-IT) and LMB-75 (Fv-IT) in mouse serum is very short, at 24 and 7 min, respectively. Several strategies have been employed to increase the serum half-life of many therapeutic protein molecules to improve their biological efficacy [42]. Based on the properties of serum albumin, which has a long half-life in serum, albumin and the protein that binds to albumin have been extensively used to improve the in vivo efficacy of many biological molecules [43,44]. The albumin binding domain (ABD) from *Streptococcus* and single domain Llama antibodies have been utilized to increase the half-life of anti-BCMA immunotoxin LMB-162 and LMb-173, respectively (Figure 4). 

LMB-162 was generated by introducing 54 amino acids containing the ABD domain from *Streptococcus* [45]. The half-life of the resulting immunotoxin in mouse serum significantly improved, although the molecule lost 2–3 fold in vitro activity. The improvement of in vivo activity of LMB-162 due to the increase in serum half-life is yet to be determined.

One of the most difficult challenges in the field of immunotoxin therapy is the elimination of immunogenicity that arises in particular from the bacterial toxin portion of the molecule, to the human immune system. Despite the positive response in the clinical setting, anti-drug antibody formation limits the use of immunotoxin during treatment. Immunogenic response to immunotoxin is in general mediated by the activation of B cells with help from T-cell dependent or independent pathways in the immune system. Several strategies have been employed to reduce the immunogenicity of RIT for therapeutic applications, including the removal of B and T cell epitopes [46,47] from the toxin domain. Several versions of modified toxins were generated by employing those strategies that retain full cell killing activity but are less immunogenic to the human immune system [48]. Various less immunogenic anti-BCMA immunotoxin molecules have been generated. LMB-92 was generated by combining the Fab fragment of mAb BM306 with a B-cell epitope-removed version of PE toxin (Figure 4). Similarly, the Fab fragment of BM306 was linked to T-cell epitope-removed toxin to generate LMB-103. Resulting immunotoxins were tested for their in vitro activity on the BCMA expressing H929 cell line (Table 1). Less immunogenic anti-BCMA immunotoxin LMB-92 and LMB-103 with B-cell (LO10R456A) and T cell (T20) epitope-removed toxin lost about 3 and 6 fold in vitro activity, respectively. Recently, an anti-BCMA immunotoxin LMB-267 was generated by combining the Fab fragment of BM306 with a modified T-cell epitope-removed toxin (T20-A494R) that retains both in vitro and in vivo efficacy similar to wild-type (LR) toxin containing immunotoxin LMB-70. In vivo efficacy and the mouse toxicity of LMB-92, LMB-103, and LMB-267 immunotoxins are yet to be determined.

## 10. Concluding Remarks

After decades of focused research by a few determined scientists, immunotoxins made the mark as viable anti-cancer treatments in the field of immunotherapy. There are two FDA approved recombinant immunotoxins for the treatment of hematologic malignancies [34,49] and several RITs are in different stages of clinical trials either as a single agent or in combination with first line chemotherapeutic drugs. Encouraging results from the ever-growing list of BCMA-targeted immunotherapies show promising anti-myeloma activity in many clinical trials. Anti-BCMA RIT will be a valuable addition to this list. It is anticipated that the anti-BCMA IT, alone and in combination with first line chemotherapeutic anti-myeloma drugs, will be evaluated in clinical studies for the treatment of multiple myeloma.

## Figures and Tables

**Table 1:** Summary table describing the properties of anti-BCMA immunotoxin variants.

Immunotoxin	Description	Molecular Weight (kDa)	IC_50_ in H929 (ng/mL)	In Vivo Efficacy (H929 SC Xenograft)	In Vivo Efficacy (H929 BM Xenograft)	Reference
LMB 70	BM306-Fab-LRggs	72	1.1	Growth Inhibition	Complete Response	[35]
LMB 107	BM306-dsFv-PE38	62	1.1	Growth Inhibition	ND	[38]
LMB 75	BM306-dsFv-LRggs	48	1.3	Growth Inhibition	Complete Response	[41]
LMB 162	BM306-dsFv-ABD-LRggs	54	3.6	Growth Inhibition	ND	[38]
LMB 173	BM306-dsFv-MSA21-LRggs	58	2.4	ND	ND	[38]
LMB 92	BM306-Fab-LO10R456A	72	3.1	Growth Inhibition	ND	[38]
LMB 103	BM306-Fab-T20	72	6.0	ND	ND	[38]
LMB 267	BM306-Fab-T20 A494R	72	1.1	ND	Complete Response	[38]

ND: Not done; SC: Subcutaneous; BM: Bone marrow.
